# The Effect of Multidirectional Loading on Contractions of the M. Medial Gastrocnemius

**DOI:** 10.3389/fphys.2020.601799

**Published:** 2021-01-18

**Authors:** David S. Ryan, Norman Stutzig, Andreas Helmer, Tobias Siebert, James M. Wakeling

**Affiliations:** ^1^Department of Biomedical Physiology and Kinesiology, Simon Fraser University, Burnaby, BC, Canada; ^2^Department of Motion and Exercise Science, University of Stuttgart, Stuttgart, Germany

**Keywords:** transverse load, ultrasound sonography, muscle architecture, contraction dynamics, muscle compression

## Abstract

Research has shown that compression of muscle can lead to a change in muscle force. Most studies show compression to lead to a reduction in muscle force, although recent research has shown that increases are also possible. Based on methodological differences in the loading design between studies, it seems that muscle length and the direction of transverse loading influence the effect of muscle compression on force production. Thus, in our current study we implement these two factors to influence the effects of muscle loading. In contrast to long resting length of the medial gastrocnemius (MG) in most studies, we use a shorter MG resting length by having participant seated with their knees at a 90° angle. Where previous studies have used unidirectional loads to compress the MG, in this study we applied a multidirectional load using a sling setup. Multidirectional loading using a sling setup has been shown to cause muscle force reductions in previous research. As a result of our choices in experimental design we observed changes in the effects of muscle loading compared to previous research. In the present study we observed no changes in muscle force due to muscle loading. Muscle thickness and pennation angle showed minor but significant increases during contraction. However, no significant changes occurred between unloaded and loaded trials. Fascicle thickness and length showed different patterns of change compared to previous research. We show that muscle loading does not result in force reduction in all situations and is possibly linked to differences in muscle architecture and muscle length.

## Introduction

Muscle force is affected by compression applied to the muscle. Research has shown that applying unidirectional transverse loads to the gastrocnemius in rats will lead to a reduction in muscle force of nearly 13% (Siebert et al., 2014a). More recently it was shown that unidirectional transverse loads have a similar effect on the human gastrocnemius where a 16% force reduction was measured at higher loads (Ryan et al., 2019; Stutzig et al., 2019). Siebert et al. (2018) showed that multidirectional transverse loading caused reductions in twitch force, but these reductions were less than for the unidirectional transverse loads. *In vitro* compression of bullfrog semimembranosus muscle showed changes in muscle force that were dependent on the length of the muscle (Sleboda and Roberts, 2019). Furthermore, finite element modeling has shown that force reduction due to compression of the muscle is dependent on both pennation angle and initial muscle length (Ryan et al., 2020).

Muscle exists as a part of a whole organism, and as such interacts with surrounding structures such as organs, bones, skin, tendons, ligaments and other muscle. In the lower leg of humans we find the tibia and fibula bones and thirteen muscles (Fukunaga et al., 1992). As such, any transverse bulging of a given muscle is going to cause it to press against either bones, other muscles, or both. These structures form barriers that impede the bulging of muscle as it contracts and relaxes or restrict muscle shape. Wick et al. (2018) found increased pennation angles and fascicle curvatures in packed compared to isolated rabbit M. soleus muscles. Muscle deforms in three dimensions during contraction. It contracts along its longitudinal axis, but due to the isovolumetric nature of muscle, it will bulge in the transverse directions as well. Azizi et al. (2017) constrained the transverse bulging of frog muscle using polypropylene tubes. This restriction to bulging caused a decrease of 50% in the power that the muscle was able to output during contraction. In rabbit muscle de Brito Fontana et al. (2018) showed that muscle force was lower when the quadriceps muscles contracted as a bundle than the summation of their individual muscle forces if they had contracted in isolation from each other.

Muscle contraction leads to changes in muscle architecture (Narici et al., 1996; Maganaris et al., 1998; Wakeling and Randhawa, 2014). Recent research has shown muscle architecture changes are affected by transverse muscle loading (Wakeling et al., 2013; Ryan et al., 2019). Muscle thickness, pennation angle, and fascicle thickness increased with muscle contraction and increased less with higher transverse loads applied to the muscle. However, it is not known if the changes in muscle architecture lead to alteration of the muscle force when the muscle is multidirectional transversely loaded.

It has been hypothesized that there is anisotropy between the changes in thickness and width of a contracting muscle (Azizi et al., 2008; Holt et al., 2016). Recent research has shown asymmetries in the bulging of muscle fascicles in humans (Randhawa and Wakeling, 2018) and in whole muscles in humans (Englund et al., 2011; Mazzoli et al., 2018) and rats (Konow et al., 2020). These asymmetries in bulging could result from asymmetries in stress distribution across the muscle (Wakeling et al., 2020). As such, differences in loading design could affect the transverse stress in the muscle, and hence the muscle architecture. Siebert et al. (2018) showed that multidirectional sling loading resulted in lower force reductions than unidirectional loading (Ryan et al., 2019; Stutzig et al., 2019). Still undetermined is whether changing from unidirectional loading to multidirectional loading will have an effect on muscle architecture.

The changes in muscle force due to transverse loading are dependent on the resting length of the muscle (Sleboda and Roberts, 2019). Muscle lengths between 0.9 and 1.1 L/L_0_ (where L and L_0_ are muscle length and optimal muscle length, respectively), showed a decrease in muscle force when compressed. The greatest reduction in muscle force was approximately 12%. Muscle length of 1.2 L/L_0_ showed an increase in muscle force when compressed, with an increase of approximately 3%. These changes in muscle force due to compression at different muscle lengths were suggested to be linked to the helical arrangement of collagen fibers in the extracellular matrix (Sleboda and Roberts, 2019; Kier, 2020). Experiments on silicone tubes wrapped in Kevlar fiber showed similar results to the bullfrog muscle experiments (Sleboda and Roberts, 2019). Larger helical angles led to reductions in force, whereas smaller angles led to increases in force. The helical angles represented extracellular matrix at different muscle lengths because the angle of the collagen fibers in the matrix reorients as the muscle changes length.

There is an indication that transverse muscle loading leads to a change in fascicle length (Ryan et al., 2019) and past research has shown that fiber force is in part determined by the length of the fiber (Ramsey and Street, 1940). Being a bi-articulate muscle (Hodson-Tole et al., 2016) the length of the medial gastrocnemius is determined by the angle of the ankle as well as the angle of the knee (Fukunaga et al., 1996, 1997; Narici et al., 1996; Herbert et al., 2002). This leads to a wide range of muscle lengths that the medial gastrocnemius can take on, with the result that it works over a range of the force–length relationship, both at rest and during activation (Maganaris, 2003; Kawakami and Fukunaga, 2006). For the current study participants are measured with their knees bent at a 90° angle in contrast to previous studies where knees were kept fully extended (Siebert et al., 2018; Ryan et al., 2019; Stutzig et al., 2019). As a result of knee angle change the whole muscle-tendon unit changes in length (Herbert et al., 2002). The medial gastrocnemius is shorter at a 90° knee angle than it is with the knee extended, though a large amount of the change in length is attributed to tendon lengthening rather than fascicle lengthening (Herbert et al., 2002). Furthermore, the torque produced by the medial gastrocnemius was shown to be lower at flexed knee angles than at extended knee angles (Cresswell et al., 1995; Maganaris, 2003). As such the knee position of the participants needs to be taken into account when comparing force measurements with previous research on *in vivo* muscle force reductions measured in humans.

As multidirectional loading resulted in less force reduction than unidirectional loading we hypothesize that muscle architecture will change but do so to a lesser degree than in our unidirectional loading study (Ryan et al., 2019). Furthermore, in contrast to previous studies examining longer muscle lengths, the shorter resting length of the muscle will alter the effects of external transverse loading. We hypothesize that the shorter length of the muscle will make it less susceptible to force reduction and architecture changes.

## Materials and Methods

Twenty young adults were recruited (sex: 3 female/17 male, age: 26.1 ± 5.0 years, height: 180 ± 6 cm, weight: 77.0 ± 10.1, BMI: 23.7 ± 2.6, physical activity per week: 6.0 ± 4.5 h). All participants were informed and gave their written consent before taking part in the study. The study was approved by the Office of Research Ethics at Simon Fraser University and University of Stuttgart.

**FIGURE 1:**
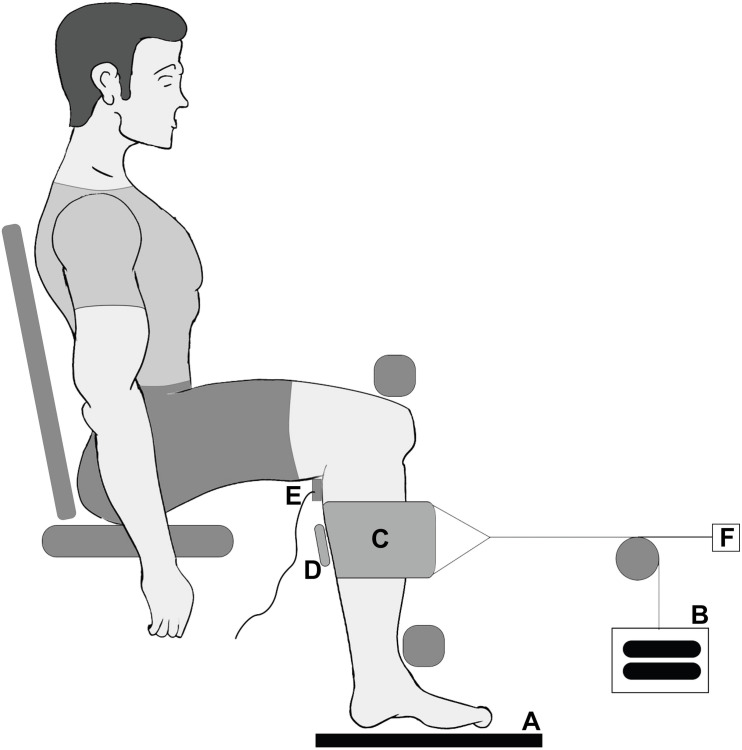
Experimental design for the collection of force data and ultrasound data. Participants were seated in a custom designed chair with their right foot placed on a force plate **(A)**. Through the use of a pully system weights **(B)** loaded the sling **(C)** which was wrapped around the calf to apply transverse loads to the muscle. Ultrasound images **(D)** were taken of the medial gastrocnemius, and the muscle was stimulated using an electrode **(E)** on the tibialis posterior nerve. A draw wire sensor was used to measure to horizontal displacement of the sling **(F)**.

A custom chair was constructed that allowed participants to sit upright with their back fully supported (Figure 1). Bars were fixed over the knees to prevent the lower leg from moving upward and in front of the shins to prevent the lower leg from moving forward during contraction. The knee and ankle angles were held at 90° angles. The right foot was placed on a force plate (AMTI OR-6, Watertown, United States) (Figure 1A) to determine twitch force throughout the trials.

To elicit contraction forces of the plantarflexors, the tibial posterior nerve (Figure 1E) was electrically stimulated using a high current stimulator (DS7AH Digitimer, Herfordshire, United Kingdom). Therefore, a cathode was placed in the popliteal fossa, and an anode was positioned on the thigh approximately 2 cm proximal to the patella. Maximal paired electrical stimuli (pulse interval 10 ms) were applied to the tibialis posterior nerve (Stutzig and Siebert, 2016).

A latex sling (420 × 150 mm) (Figure 1C) was wrapped around the calf at the height of the gastrocnemii and attached to a cable and pulley system. The sling was kept in place for all trials, and during weighted trials either 5 or 10 kg were attached to the pulley system (Figure 1B). The horizontal displacement of the sling was recorded (sample rate: 1,000 Hz) using a draw wire sensor (SX50, WayCon, Taufkirchen, Germany) (Figure 1F).

A linear ultrasound probe (Echoblaster 128, Telemed, Lithuania) (Figure 1D) was positioned superficial to the sling to scan through to the medial gastrocnemius, providing a 65 mm field of view with a 50 mm scanning depth (604 × 515 pixels; 80 Hz). The ultrasound probe was centered on the medial gastrocnemius in both the anterior–posterior and medial–lateral directions. The probe surface was kept flat on the surface of the sling and aligned so that complete fascicles appeared in the scanning plane.

The participants performed a warm-up that consisted of 10 repetitions of walking a set of stairs, three sets of rope skipping (20 skips per set), and 10 calf raises. Participants were then seated and secured in the measurement chair, and the stimulation electrodes were placed. The stimulation current was increased by 10 mA increments until the measured maximal double twitch force showed a plateau. The second stimulation current at the plateau region was used throughout the trials.

The study consisted of four conditions: pre-test, post-test, and two loaded condition. Pre- and post-test had no load applied to the sling. 5 and 10 kg weights loaded the sling for the loaded trials and were applied in a random order. Three double twitches were evoked every 10 s in each condition.

The maximum twitch force between the pre- and post-test were compared to determine reliability of the performed trials. The set of trials was deemed unreliable when differences between the maximum twitch force between pre- and post-test were greater than 5%, as something within the set-up might have changed to cause the difference in force. Unreliable trials were removed from the data set.

**FIGURE 2:**
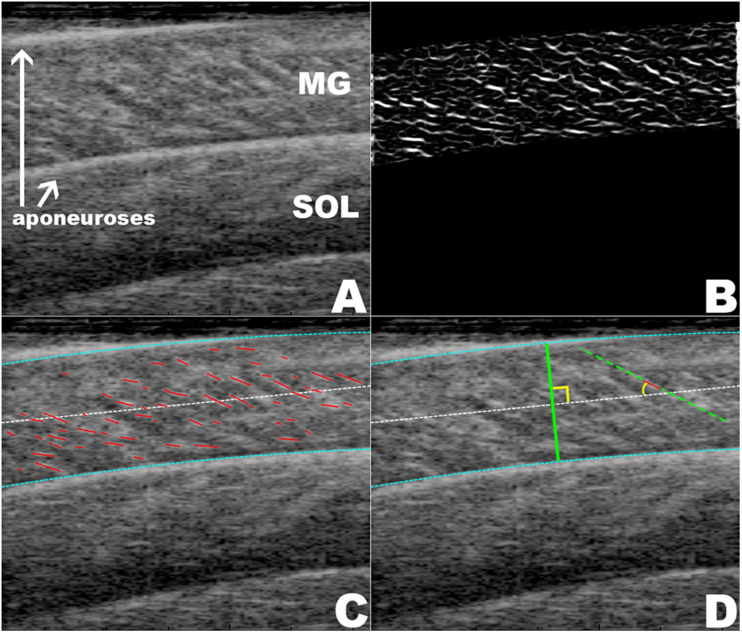
Depictions of the ultrasound data analysis. **(A**) A raw ultrasound frame with medial gastrocnemius (MG), soleus (SOL), and aponeuroses shown. **(B)** The result of the multiscale vessel enhancement filter applied to the ultrasound frame. **(C)** The digitization of the aponeuroses (blue) and the fascicle lines (red). **(D)** Shown are the measurement of the muscle thickness and the pennation angle of a fascicle.

The ultrasound data (Figure 2) were analyzed for a series of frames centered around the maximum twitch force in four steps following our previous procedures (Ryan et al., 2019): manual digitization, multiscale vessel enhancement filtering (Frangi et al., 1998), Hough line detection (Hough, 1962; Duda and Hart, 1972; Illingworth and Kittler, 1988), and Fourier transformation (Wakeling and Randhawa, 2014). These steps allowed us to quantify the muscle thickness, pennation angle, and the spacing of the fascicular stripes that we term the fascicle thickness. Fascicle lengths were estimated by dividing muscle thickness by the sine of the pennation angle. All raw data were filtered using a Gaussian filter before analysis.

In each frame of the ultrasound data the aponeuroses were manually segmented using a custom written Mathematica code (Wolfram Mathematica 11, Oxfordshire, United Kingdom). The muscle thickness was calculated as the mean distance between the aponeuroses. Frames in which the muscle was isolated had the multiscale vessel enhancement filter applied to them using scales 1, 2, and 3 (Frangi et al., 1998; Rana et al., 2009; Namburete et al., 2011). Hough line detection was applied to the filtered gastrocnemius regions (Hough, 1962). The filtered image was binarized to produce a black and white image. For each morphological component in the binarized image we applied the Hough transform to determine the best fit line. From each of these lines we then determined the angle relative to the *x*-axis (long axis of the image). As the fascicles ran from top left to bottom right of each image only negative angles were representative of fascicles and positive angles were discarded. Similarly, any angles smaller than 5° were discarded as they most likely represented blood vessels running through the muscle. The final pennation angle was calculated by taking the mean angle of all lines relative to the mean direction of the aponeuroses, which was assumed to be the line of action of the muscle.

For each isolated and enhanced frame the wavelength of the fascicles in the muscle belly was determined using a 2D-Discrete Fourier transform (Wakeling and Randhawa, 2014; Ryan et al., 2019). The transform was applied to find the transverse wavelength across the muscle fascicles. Stripes in the ultrasound images were considered to lie in the same direction as the muscle fascicles. As the fascicles dilated, the spacing of these stripes got wider; thus their transverse wavelength was proportional to the fascicle thickness.

The pennation angle, muscle thickness, fascicle thickness, and fascicle length were determined for passive loaded muscle before muscle activation. This was done for all four trials. Statistical analyses were performed to determine significant differences between the passive states of the four trials for all muscle architecture measurements. The maximum, for pennation angle and muscle thickness, and the minimum, for fascicle thickness and fascicle length, were determined from data spanning 0.5 s centered around peak twitch force. These maxima and minima were determined for all four trials. Statistical analyses for all architecture measurements were performed to determine significant differences between either the maxima or minima of the four trials. All the statistical tests were calculated using a one-way repeated measures ANOVA in IBM SPSS Statistics (Version 25 IBM Corp., Armonk, NY). Subjects were considered random and twitches between individuals were considered to be independent of one and other. Differences between trials were determined with a *post hoc* Bonferroni test. Tests were considered significant when *p* < 0.05.

For all four muscle architecture measurements, the value for passive loaded muscle and either maximum or minimum were compared to one and other to determine if muscle architecture significantly changed with muscle activation. Significant differences were calculated using independent samples *t*-tests. Tests were considered significant when *p* < 0.001. A smaller *p*-value was used for these *t*-tests compared to the one-way repeated measures ANOVAs to allow for the Bonferroni correction for multiple comparisons.

## Results

**FIGURE 3:**
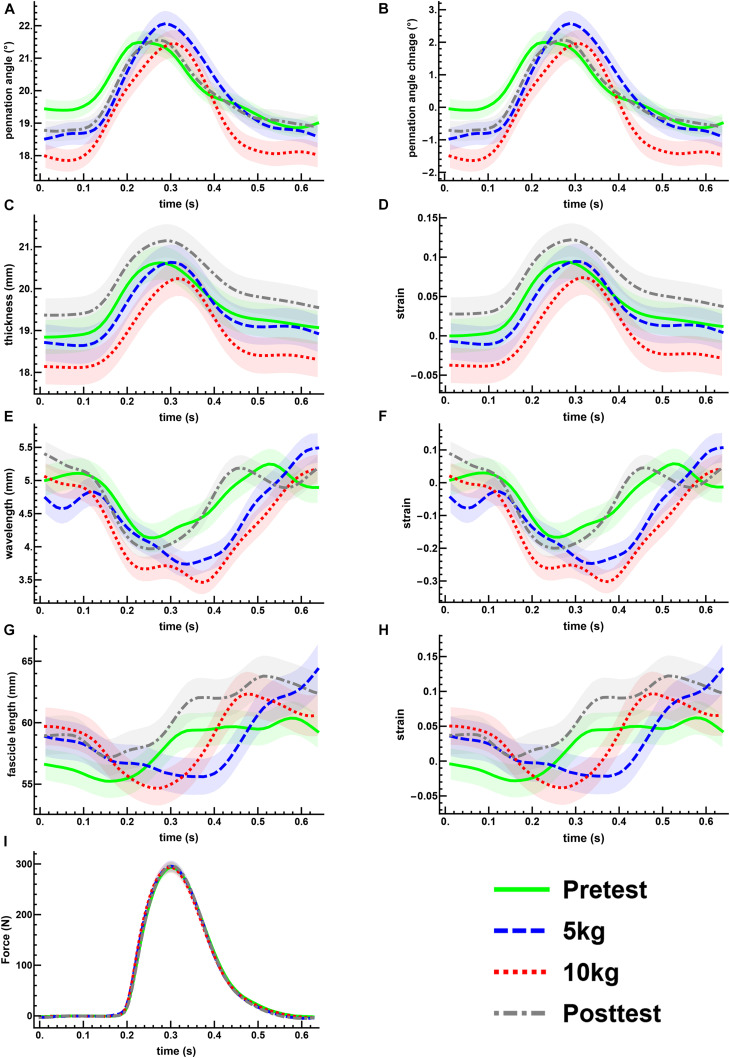
The mean changes in muscle architecture and force measured during muscle twitches. The mean and standard error for all measured twitches are plotted for the pre- (green, solid) and post-test (gray, dot-dashed), and the 5 kg (blue, dashed) and 10 kg (red, dotted) loaded trials. Transient increases are shown for the pennation angle **(A,B)** and muscle thickness **(C,D)**. The transverse wavelength **(E,F)** and fascicle length **(G,H)** show transient decreases. The measured twitch force **(I)** shows an increase with muscle activation.

**FIGURE 4:**
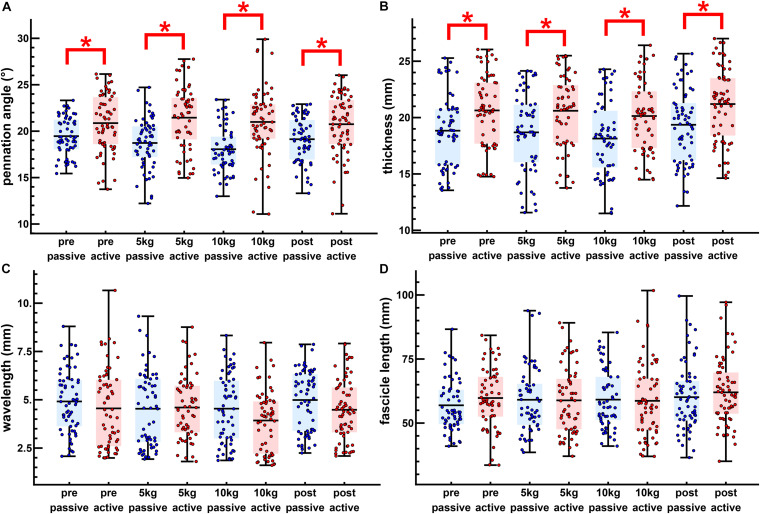
The passive loaded and peak active loaded values for all twitches. Passive (blue) and active (red) values are shown for the pennation angle **(A)**, muscle thickness **(B)**, transverse wavelength **(C)**, and fascicle length **(D)**. The dots show individual twitches with the boxes showing the mean and span from the 0.25 to the 0.75 quantile. Significant differences are shown between passive and active states (^∗^*p* < 0.05).

Excitation of the posterior tibialis nerve resulted in twitch contractions of the triceps surae muscles. This was shown by the transient increase in force (Figure 3I). During each twitch the GM increased in muscle thickness (Figures 3C,D), pennation rotated to a greater angle (Figures 3A,B), and fascicle thickness (wavelength) and fascicle length decreased (Figures 3E–H, 4).

The maximal twitch force remained unaltered in all conditions (Figure 3I). There was a little effect of the transverse load on the muscle architecture. For the passive state (before each twitch), the transverse load resulted in a significant decrease in the pennation angle [*F*_(3_, _180)_ = 11.4, *p* < 0.001, η^2^*_*p*_* = 0.160] (Figures 3A,B) and the muscle thickness (Figures 3C,D), but not in the fascicle length or transverse wavelength (Figures 3C–H). The peak thickness reached during the twitch was significantly lower in the loaded contractions (Figures 3C,D), but there was no significant effect of the transverse load on the peak pennation, fascicle length or transverse wavelength (Figures 3A,B,E–H).

**FIGURE 5:**
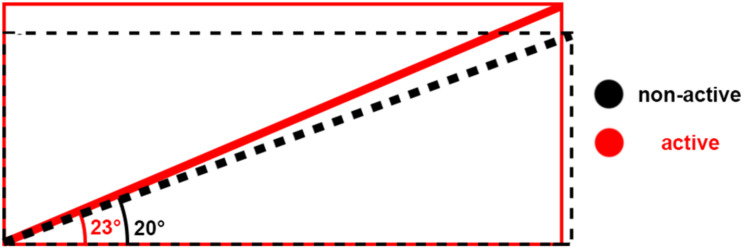
Schematic of architecture changes between non-active (black) and active (red). The schematic shows that when muscle shortens, bulges, and the pennation angle increases, the fiber length may remain at the same length.

Muscle thickness (*t*_124_ = −3.18, *p* < 0.005, *t*_120_ = −3.15, *p* < 0.005, *t*_120_ = −3.42, *p* < 0.005, *t*_130_ = −3.26, *p* < 0.005, pretest, 5, 10 kg, post-test, respectively), and pennation angle (*t*_124_ = −3.0, *p* < 0.005, *t*_120_ = −4.90, *p* < 0.001, *t*_120_ = −5.21, *p* < 0.001, *t*_130_ = −3.28, *p* < 0.005, pretest, 5, 10 kg, post-test, respectively), significantly increased between the passive and active states of each twitch. Fascicle wavelength and fascicle length were shown to change during activation (Figures 3E–H), however, *t*-tests showed no significant differences between the passive and active states (Figure 4). As such it can be said that muscle thickness and pennation angle changed with muscle activation, but fascicle thickness and fascicle length remained unchanged with muscle activation (Figure 5).

Significant differences were mainly found for the pennation angle and muscle thickness. Passively loaded muscle showed significant differences between the pretest and loaded trials for pennation angle and between the post-test and the loaded trials for the muscle thickness. The change from passive to actively loaded muscle was significantly different for the pretest and loaded trials for the pennation angle. Changes for muscle thickness were not significant although actively loaded muscle showed differences between the post-test and the loaded trials. Changes between the pretest and loaded trials were significant between the pretest and the loaded trials. Changes in fascicle length were not discernable from variability between the pre-test and post-test.

During muscle activation the bulging of the muscle caused the transverse load to increase in height (lifting height). The lifting height of the transverse load was measured during each twitch for all the trials. The mean lifting heights for the unloaded pre- and post-test were 5.04 ± 1.52 and 5.15 ± 1.59 mm, respectively. For the 5 kg trials the mean lifting height was 12.38 ± 3.42 mm and for the 10 kg trials the mean was 15.89 ± 4.05 mm. No significant differences were found between the mean lifting height of the pre- and post-test. The unloaded trials were significantly different from the loaded trials, and the mean lifting heights of the 5 and 10 kg trials were significantly different from each other (*p* < 0.05).

The lifting work of the muscle was estimated for the 5 kg and 10 kg loaded trials. Lifting work was estimated at 2.48 × 10^3^ and 6.36 × 10^3^ J m^–3^ for the 5 and 10 kg trials, respectively, when expressed per volume of the MG muscle (243.7 cm^3^, Fukunaga et al., 1992).

## Discussion

In this study we found no change in twitch force when the human gastrocnemius muscle was transversely loaded with a compression sling. This finding is different from previous studies where decreases in force due to muscle loading have been reported in rat (Siebert et al., 2014a, b) and human studies (Siebert et al., 2018; Ryan et al., 2019; Stutzig et al., 2019). A major difference in this current study was that the muscle was held at a shorter length: in this study the mean fascicle length was 60 mm, compared to our previous length of 70 mm (Ryan et al., 2019). However, recent studies have shown that compression-related reductions in muscle force may be exacerbated at shorter muscle lengths (Sleboda and Roberts, 2019), and thus the differences in our study may be due to a complex interaction of different factors during contraction: fascicle length, pennation angle, and direction of loading.

In the endomysium the collagen fibers were shown to have an average angle of 59° which got lower in lengthened muscle (34°) and higher in shortened muscle (73°) (Purslow and Trotter, 1994). Physical muscle models showed that a helical fiber arrangement with a low angle reacted to compression by muscle shortening, whereas with a high angle compression caused muscle lengthening (Sleboda and Roberts, 2019). According to our data transverse loading of passive muscle did not cause a change in fascicle length. The assumption is therefore that in our current experiment the fascicles were working over the same range of the force–length relationship for both unloaded and loaded muscle. As a result, we would expect no change in muscle force, which is what the current results showed. In previous studies (Ryan et al., 2019; Stutzig et al., 2019) muscle was kept at a longer length. In longer muscle, transverse loading would lead to shortening of the fascicles, assuming that the helical angle of the extracellular matrix was lower than 59°. This would explain the force reduction as the fascicles would be working lower on the ascending limb of the force-length relationship when active (Kawakami and Fukunaga, 2006). However, fascicle length was not measured for unloaded conditions in previous studies.

As fascicle length decreases it is expected that fascicle thickness would increase due to the isovolumetric properties of fascicles. However, the data show a decrease in fascicle thickness. It is possible that the decrease in both length and thickness is compensated by an increase in fascicle width, which was not measured during these experiments. Muscle measurements in the medial gastrocnemius of rats showed that widthwise strains were approximately twice that of thickness strains (Konow et al., 2020). Raiteri et al. (2016) showed significant differences between the changes in thickness and width of the tibialis anterior. A similar difference in strains could be possible in muscle fascicles. It has been shown that fascicle deformation can be transversely anisotropic (Randhawa and Wakeling, 2018). Therefore, the difference between the changes in fascicle thickness and fascicle width could be possible.

Azizi et al. (2017) used tubes to constrain muscle bulging and this can be considered similar to multidirectional loading. Azizi et al. (2017) kept muscle close to optimal muscle length whereas our current study used muscle lengths shorter than optimal length. As they measured a reduction in muscle performance where we did not, this might indicate that muscle length plays a role in the effect caused by muscle loading.

Cresswell et al. (1995) found that plantar flexor torque was lower when the knee was flexed compared to the knee being extended. With the knee flexed at 60°, plantar flexor torque was 60% of the torque found with the knee fully extended. The possibility remains that because the gastrocnemii are producing a lower force (due to the force–length relation), they were less affected by transverse loading which could contribute to the lack of force reduction found in this study.

One consequence of short muscles are shorter fascicles and greater pennation angles. Shortening of fascicles leads to bulging in the transverse directions due to their isovolumetric characteristics (Abbott and Baskin, 1962; Baskin and Paolini, 1967) which would result in increased pennation angle (Randhawa and Wakeling, 2018). This was reflected in the pennation angle measurements of the current study which were close to 20°compared to about 10° in previous studies examining longer muscle lengths (Ryan et al., 2019; Stutzig et al., 2019). It should be noted that lower pennation angles would not only be due to longer fascicle lengths but likely also due to gravity compressing the muscle because participants were lying in a prone position for the previous study (Ryan et al., 2019). Muscle with a pennation angle lower than 15° tends to thicken with muscle activation whereas muscle with a pennation angle higher than 15° tends to get thinner with muscle activation (Wakeling et al., 2020). In the previous studies (Ryan et al., 2019; Stutzig et al., 2019) muscle would tend to get thicker because of the lower pennation angle, which was shown in the muscle thickness measurements. This tendency to expand against the direction of the transverse load could contribute to the reduction in twitch force that was measured. In our current study the muscle would tend to get thinner particularly in the 10 kg condition because of the higher pennation angle. This would cause it to not expand against the transverse load and would not contribute to any force reductions. However, the muscle thickness measurements of the current study showed an increase in thickness with muscle activation which goes against supporting this argument, albeit this increase in thickness was less (2 mm) than in previous studies (5 mm: Ryan et al., 2019; Stutzig et al., 2019).

For the unidirectional loaded study (Ryan et al., 2019) the same 10 kg load was used on a smaller surface area compared to this multidirectional loading, resulting in a higher pressure. Despite the same amount of transversal loading, unidirectional loading results in a reduction of muscle force (Ryan et al., 2019; Stutzig et al., 2019), but multidirectional loading not (Figure 3I). In contrast, experiments on isolated rat muscles showed that when applying a constant transversal load, force reduction was independent of the transversal pressure (Siebert et al., 2016). In this study muscle shape and thus presumably muscle architecture remained almost unchanged for different unidirectional pressures induced by equally loaded plungers with different contact areas. However, higher pressure in unidirectional transversal loading studies (Ryan et al., 2019; Stutzig et al., 2019) resulted in greater muscle deformations and consequently greater changes in resting pennation and muscle thickness compared to the multidirectional transversal loading applied in the present study (Figure 4), which might lead to different influences on force generation. Furthermore, the load applied by the sling would not solely be on the medial gastrocnemius but shared between the medial and lateral gastrocnemius and the rest of the lower leg. As such the 10 kg load of the unidirectional block loading is not fully comparable to the 10 kg load of the multidirectional sling loading.

The absence of higher levels of multidirectional transverse loading was one of the limitations in this study. The loads were chosen according to loads used in previous studies (Ryan et al., 2019; Stutzig et al., 2019), however, the differences in the experimental design could have altered the load needed to show significant effects of loading on the muscle.

The reliability of the ultrasound remains an issue because changes in the position of the ultrasound probe can lead to errors (Bolsterlee et al., 2016). Furthermore, in 2D ultrasound imaging the assumption is made that the fascicles run parallel to the plane of the image. However fibers are known to be curved throughout the muscle (Rana et al., 2013, 2014; Papenkort et al., 2020). As such the muscle architecture measurements made are an approximation of the actual values. Our analysis of the baseline variability from consecutive twitches suggested that the changes in muscle architecture were reliable within a threshold of one standard deviation, apart from for the fascicle length. The fascicle length presented here is a derived parameter based on both muscle thickness and pennation angle, and so will be susceptible to errors from both these measures. Variations in fascicle length with compression were within the variation of lengths between the pre- and post-tests, and so we were unable to detect significant effects of the external load on fascicle length.

The discrepancy between the number of males and females in the experimental group might be an issue. To test for this effect we additionally tested for differences in mean maximum force for the male participants only. No significant differences were found similar to when females were included in the experimental group.

## Conclusion

In conclusion, previous studies showed that external unidirectional transverse loading of the medial gastrocnemius reduced muscle force. However, using an alternative design inducing multidirectional transversal muscle loading we observed no reduction in force. This is likely due to lesser effects of multidirectional transversal loading on muscle deformation and architecture compared to unidirectional transversal loading as well as a shorter muscle length and higher pennation angles as a result of participants being seated with their knee flexed 90°.

## Data Availability Statement

The raw data supporting the conclusions of this article will be made available by the authors, without undue reservation.

## Ethics Statement

The studies involving human participants were reviewed and approved by the Office of Research Ethics at Simon Fraser University and University of Stuttgart. The patients/participants provided their written informed consent to participate in this study.

## Author Contributions

DR, NS, AH, TS, and JW contributed to the study design, contributed to the ultrasound and force measurements, and analysis. All authors contributed to the article and approved the submitted version.

## Conflict of Interest

The authors declare that the research was conducted in the absence of any commercial or financial relationships that could be construed as a potential conflict of interest.

## Appendix

Here, we show the values for the resting passive and peak active state, as well as the change between the two states (Table A1). Values are given for the pennation angle, muscle thickness, transverse wavelength, and fascicle length. The data shown in Table A1 matches the data shown in Figure 3.

**TABLE A1:** Comparison of the resting passive and peak active values between trials.

	Pretest	5 kg	10 kg	Post-test	Significance
**Pennation angle (°)**					
Passive	19.4 ± 2.0	18.7 ± 2.7	18.1 ± 2.4	19.2 ± 2.4	*,†, +, @
Active	20.9 ± 2.1	21.5 ± 3.4	21.0 ± 3.7	20.7 ± 3.3	
Change	2.7 ± 1.5	3.8 ± 1.4	3.8 ± 1.5	3.1 ± 1.4	*,†,‡

**Muscle thickness (mm)**					
Passive	18.9 ± 3.2	18.7 ± 3.5	18.1 ± 3.3	19.1 ± 3.2	†,‡, +, @
Active	20.7 ± 3.2	20.6 ± 3.3	20.1 ± 3.1	21.0 ± 3.2	†,‡, +, @
Change	1.8 ± 0.7	2.0 ± 0.9	2.2 ± 1.4	1.9 ± 0.7	

**Transverse wavelength (mm)**					
Passive	4.9 ± 1.6	4.5 ± 1.9	4.5 ± 1.8	5.0 ± 1.5	
Active	4.6 ± 2.0	4.6 ± 1.6	3.9 ± 1.6	4.5 ± 1.6	±
Change	1.4 ± 0.7	1.9 ± 1.0	2.0 ± 1.2	1.7 ± 0.9	*,†

**Fascicle length (mm)**					
Passive	57.2 ± 10.2	59.1 ± 12.6	59.2 ± 11.3	59.2 ± 12.6	†
Active	60.0 ± 11.9	58.9 ± 13.0	58.7 ± 14.5	61.8 ± 13.2	
Change	7.0 ± 2.9	8.4 ± 3.9	8.6 ± 5.5	8.6 ± 3.8	|

*Means and standard deviations are given for the pennation angles, muscle thicknesses, fascicle wavelengths, and fascicle lengths. Significances are indicated between pre-test and 5 kg (^∗^), pre-test and 10 kg (†), pre-test and post-test (|), 5 kg and 10 kg (±), 5 kg and post-test (‡), and 10 kg and post-test (+).*
